# Spatial Regulation of Trait–Environment Interactions Shapes Plant Community Functional Patterns in a Tropical Forest

**DOI:** 10.1111/jvs.70169

**Published:** 2026-08-21

**Authors:** Rajapandian Kanagaraj, Warren Y. Brockelman, Thorsten Wiegand, Anuttara Nathalang, Jie Yang, Nitin K. Tripathi, Wirong Chanthorn

**Affiliations:** 1 Department of Environmental Technology and Management, Faculty of Environment, Kasetsart University, Bangkok, Thailand; 2 Department of Ecological Modelling, Helmholtz Centre for Environmental Research–UFZ, Leipzig, Germany; 3 National Biobank of Thailand, National Science and Technology Development Agency, Pathum Thani, Thailand; 4 Institute of Molecular Biosciences, Mahidol University Salaya, Nakhon Pathom, Thailand; 5 German Centre for Integrative Biodiversity Research (iDiv), Halle-Jena-Leipzig, Leipzig, Germany; 6 CAS Key Laboratory of Tropical Forest Ecology, Xishuangbanna Tropical Botanical Garden, Chinese Academy of Sciences, Kunming, China; 7 Department of Information & Communication Technologies, School of Engineering and Technology (SET), Asian Institute of Technology, Pathum Thani, Thailand

**Keywords:** community assembly, determinants of community functional traits, environmental filtering, INLA-SPDE approach, soil nutrients, spatial modeling, topographic water availability

## Abstract

**Question:**

Inferring community assembly mechanisms requires an understanding of the responses of plant communities to environmental gradients and spatial processes. In a tropical forest, we asked: (1) What environmental gradients are associated with variation in community functional composition and diversity? (2) How does explicitly accounting for spatial structure influence inference about these environmental effects? and (3) To what extent can the observed trait–environment relationships be explained by environmentally structured species turnover (i.e., changes in species composition along environmental gradients)? **Location:** A 30-ha Mo Singto old-growth tropical forest dynamics plot in Khao Yai National Park, Thailand.

**Methods:**

We applied a spatially explicit hierarchical Bayesian modeling (INLA-SPDE) framework to data from 135,000 woody plants, measuring eight functional traits and 24 environmental variables covering topography, soil nutrients, and canopy structure. The model estimates environmental effects while accounting for spatial autocorrelation in trait distributions. We further used null model tests to evaluate question (3).

**Results:**

Spatially explicit models identified topographic water availability (TWI) as the main environmental gradient associated with community-level trait patterns, aligning functional composition along the trade-off between acquisitive and conservative resource-use strategies. Inferences for several environmental variables changed substantially after accounting for spatial auto-correlation. Null model analyses indicated that most trait–environment relationships in the Mo Singto forest plot were consistent with environmentally structured species turnover, with only limited evidence for additional trait–environment associations, as shown by tree height.

**Conclusions:**

Our results indicate that when traits are represented at the species level, smaller-scale community-weighted mean (CWM) patterns are primarily explained by species replacement along environmental gradients. Our study also demonstrates that high-resolution, fully stem-mapped forest plots provide strong power to detect environmental signals and reveal spatially structured patterns in community functional composition. By combining spatially explicit models with null model inference, we show that robust interpretation of CWM trait–environment relationships requires jointly accounting for spatial structure and species turnover, which provides a clear framework for understanding how environmental gradients shape community functional patterns.

## 1 Introduction

Ecologists have long been intrigued by the complex interplay between environmental factors and plant functional traits (Díaz and Cabido 2001). Plant trait combinations observed at the community level are expected to reflect the distinct ecological strategies of species to cope with abiotic and biotic factors (de Bello et al. 2021a). Local environmental factors such as soil nutrients, availability of light and water, and topography influence the structure and functional composition of plant communities (e.g., Possen et al. 2011; Katabuchi et al. 2012; Liu et al. 2014; Oddershede et al. 2015; Pinho et al. 2018; Salas-Morales et al. 2018; Wang et al. 2022). For example, water availability, which varies greatly on a fine scale, is an important local factor shaping plant functional patterns and community composition (Silvertown et al. 1999; Araya et al. 2011; Liu et al. 2014; Oddershede et al. 2015). Resource-rich fertile areas support species with a predominant resource acquisition strategy that enables them to capture resources quickly and achieve high growth rates. In contrast, species in nutrient-poor environments are expected to use more conservative resource-use strategies by increasing their competitive ability (Wright et al. 2004; Poorter and Bongers 2006; Reich 2014). One approach to relate traits to resource-use strategies is to upscale the traits of individual plants to local community functional composition, often measured using community-weighted mean (CWM) traits (Garnier et al. 2004) and functional dispersion (FDis; Laliberté and Legendre 2010), and to relate them to environmental gradients.

A high diversity of traits (i.e., high values of FDis) reflects greater variety of resource use and growth strategies in the community, which can facilitate species coexistence and influence ecosystem functioning, such as productivity (Bialic-Murphy et al. 2024). According to the resource capture hypothesis, differences in functional traits among organisms that facilitate complementarity, such as niche partitioning or positive species interactions, lead to more effective acquisition of available resources (Cardinale et al. 2012). In tropical dry forests, topographic variation in water availability, reflected in soil moisture and wetness measures (Sørensen et al. 2006), can promote species diversity through niche differentiation (Balvanera et al. 2011; Punchi-Manage et al. 2013; Salazar Villegas et al. 2023). We expect that tree species adapt to available resources, such as water, light, and nutrients by employing distinct life history strategies which should be mirrored by the community functional composition.

However, revealing the relationships between species traits and environmental variability is complicated by a number of effects caused by species composition that may lead to Type I error (Pillar and Duarte 2010; Peres-Neto et al. 2017; Zelený 2018; Duarte et al. 2018; Lepš and de Bello 2023). First, plants in close proximity may share similar trait values, not only due to environmental filtering, but also because of dispersal limitation (Hubbell 2001) or regional abundance patterns. This results in trait values being more similar among nearby individuals than among those farther apart, leading to spatial autocorrelation in CWMs and low β-diversity (Zelený 2018). More generally, spatial patterns in plant distributions arise from a combination of environmental heterogeneity and biotic factors such as species interactions (competition, predation, and mutualism), dispersal limitations, and local adaptations (Seidler and Plotkin 2006; McFadden et al. 2019). Consequently, these processes contribute to spatial patterns in the distribution of plant traits. We therefore use the term “spatially structured processes” as an operational umbrella for processes that generate spatial dependence in trait distributions, including unmeasured environmental variation, dispersal limitation, species interactions, and historical factors. Models that ignore spatial processes may overlook important drivers of plant distribution across scales (Dirnböck and Dullinger 2004), and failing to account for spatial structure can inflate the statistical significance of environmental variables (Kühn 2007).

Second, because CWMs are calculated from species abundances and fixed species-level trait values, environmental gradients can influence community-level trait patterns indirectly through their effects on species composition (Peres-Neto et al. 2017; Zelený 2018). If environmental heterogeneity leads to variation in species occurrences and abundances across sites, apparent trait–environment relationships may emerge as a consequence of species replacement rather than from direct effects of the environment on trait values. As a result, significant relationships between CWMs and environmental variables can arise even when species traits are randomly assigned to species (Zelený 2018). We therefore use the term “environmentally structured species turnover” to refer to this pathway. Thus, community functional patterns along environmental gradients may arise through multiple, nonexclusive processes, where observed trait–environment relationships can reflect either species turnover or additional trait–environment associations beyond compositional change (Kraft et al. 2008; Lepš et al. 2011; Swenson et al. 2012; Peres-Neto et al. 2017; Zelený 2018). Distinguishing these alternatives is essential for correctly interpreting trait–environment relationships, particularly in spatially structured systems where residual spatial autocorrelation can obscure underlying environmental signals.

Third, when one or a few dominant species change their abundance within a plot, their trait values can strongly influence the observed variation in CWMs, even if the traits themselves are not causally related to environmental variation (Lepš and de Bello 2023). The latter two issues, which arise from the dependence of CWMs on species composition, can be addressed using null model approaches that evaluate whether observed trait–environment relationships exceed expectations based on species turnover alone (Peres-Neto et al. 2017; Zelený 2018; Duarte et al. 2018; Lepš and de Bello 2023), whereas spatial autocorrelation (issue one) is accounted for separately by using spatial models.

Studies examining spatial patterns and processes in community functional attributes, such as the CWM and FDis of traits, have employed methods such as the Mantel test (e.g., Pinho et al. 2018), autoregressive models (e.g., Liu et al. 2014), or mixed-effects models with discrete random effects (e.g., Aguirre-Gutiérrez et al. 2022). However, space is inherently continuous, especially at local scales, and recent statistical advances have allowed for a more flexible representation of spatial variation within a study area. The integrated nested Laplace approximation—stochastic partial differential equations (INLA-SPDE) approach (Rue et al. 2009; Lindgren and Rue 2015)—offers a sophisticated method for modeling continuous spatial fields within a Bayesian mixed-modeling framework. This approach improves upon traditional mixed modeling by providing more accurate estimates of standard errors and degrees of freedom, reducing the risk of inflated Type I error rates (Zuur et al. 2017), and facilitating the detection of true underlying spatial patterns or environmental effects.

The INLA-SPDE approach is particularly well suited for analyzing fine-scale spatial variation in community functional attributes in fully stem-mapped forest plots, where the spatial location of each tree above a certain size is recorded. Such high-resolution spatial data allow trait–environment relationships to be examined at spatial scales where ecological processes operate locally. This framework explicitly accounts for spatial autocorrelation in trait distributions while estimating the effects of measured environmental variables, aligning with the general rationale of partitioning environmental and spatial components by Borcard et al. (1992). However, unlike the spatial eigenfunction approaches (e.g., PCNM or MEM), the spatial dependence is modeled as a continuous spatial process, which allows spatial autocorrelation to be represented across multiple spatial scales. However, detecting spatial autocorrelation does not necessarily reveal the processes generating it (Diniz-Filho et al. 2012). When major environmental predictors are included in the model, the remaining spatial structure may reflect spatially structured processes that are not explicitly measured such as dispersal limitations, biotic interactions, historical legacies, or unmeasured environmental variation (McFadden et al. 2019). In this way, spatially explicit models provide a framework for identifying residual spatial patterns in trait distributions, while providing unbiased effects of measured environmental variables.

In this study, we combine spatially explicit INLA-SPDE modeling with null model testing to disentangle the contributions of environmental effects, spatially structured (biotic) processes, and species composition in shaping community functional composition. This framework allows us to distinguish between spatially structured patterns and environmentally driven variation while accounting for spatial autocorrelation. Using data from a fully stem-mapped 30-ha old-growth tropical forest dynamics plot in Thailand, we address three main questions: (1) What environmental gradients are associated with variation in community functional composition and diversity? (2) How does explicitly accounting for spatial structure influence inference about these environmental effects? and (3) To what extent can the observed trait–environment relationships be explained by environmentally structured species turnover, as opposed to direct trait–environment associations beyond species compositional change? We hypothesize that environmental gradients significantly structure community functional composition, but that these effects are partly confounded by spatial structure. We further hypothesize that most trait–environment relationships will arise from environmentally structured species turnover, and only a subset of traits should exhibit additional environmental associations beyond compositional change. By combining spatially explicit modeling with null model inference, our approach allows us to evaluate whether environmental effects identified in statistical models reflect species replacement or require additional trait–environment structure.

## 2 Materials and Methods

### 2.1 Study Plot and Trait Data

We conducted the study at a 30-ha (500 × 600 m) fully stem-mapped Mo Singto ForestGEO plot (Brockelman et al. 2017), located at 101°22′E 14°26′N in Khao Yai National Park, Thailand. The plot is located in an intact tropical seasonal evergreen forest at an altitude of 720–815 m above sea level, with an average annual precipitation of 2200 mm, an average temperature of 22.4°C (19.4°C–22.4°C), and a dry season of 4–6 months (Brockelman et al. 2017). Based on the ForestGEO standard protocol, the plot was divided into 20 m × 20 m quadrats and all trees with DBH ≥ 1 cm were tagged, mapped, identified to species level, and their DBH was measured. The tree census data used in this study were collected between 2015 and 2017, and details of the census are provided in the Supporting Information (Appendix S1).

**Figure 1:**
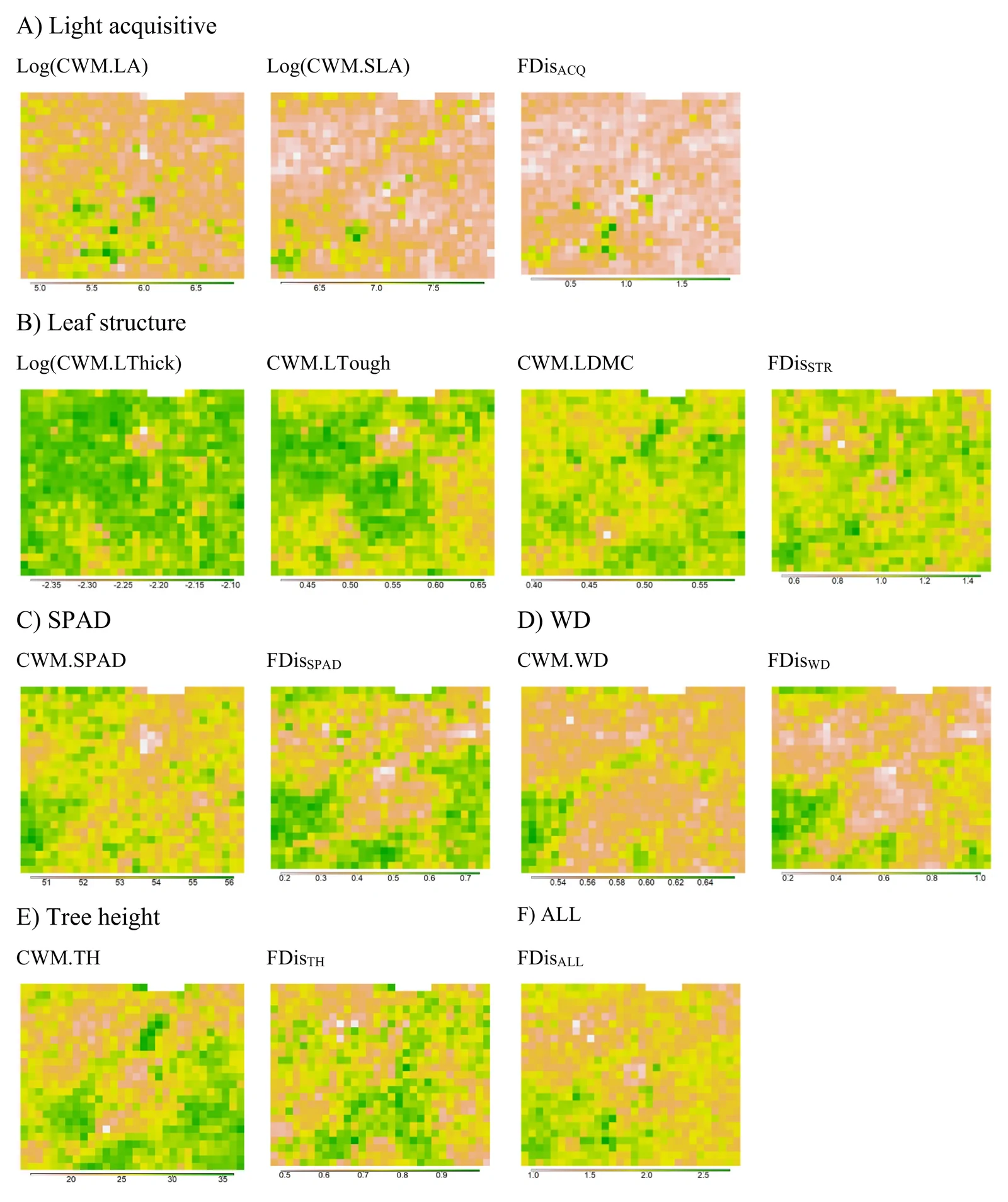
The distribution of community functional attributes, specifically the community-weighted mean (CWM) and functional dispersion (FDis), is presented at a spatial scale of 20 m × 20 m within an old-growth tropical forest in Thailand. For each 20-m subplot, the abundance-weighted mean trait value (CWM; Garnier et al. 2004) and functional dispersion (FDis; Laliberté and Legendre 2010) were computed. The dimensions of leaf acquisitiveness and leaf structure were identified using the principal component analysis (PCA) axes of all traits.

Eight plant functional traits were used (Figures 1 and S1), including leaf area, specific leaf area, leaf thickness, leaf toughness, leaf dry matter content, leaf greenness, wood density, and maximal tree height. The methodology and protocol for gathering these trait data have been documented in detail by Pothasin et al. (2022) (see Appendix S3 for a summary). We performed a principal component analysis (PCA) on the standardized trait data. The significant axes were identified using Kaiser’s criterion (eigenvalues > 1). Traits were grouped by their contribution to these PCs (high/medium/low, based on the top, middle, and bottom 33% of the total contributions) and independently classified into functional groups based on the established leaf economics theory (Figure S1). The *Leaf acquisitive* dimension comprised leaf area (LA, cm^2^) and specific leaf area (SLA, cm^2^/g), whereas the *leaf structural* dimension included leaf thickness (LThick, mm), leaf toughness (LTough, newtons), and leaf dry matter content (LDMC, mg/g). The remaining traits were interpreted separately (others: leaf greenness measured by the SPAD chlorophyll meter (SPAD, unitless), wood density (WD, g/cm^3^), and maximal tree height (TH, m)).

We divided our study plot into 20 m × 20 m subplots and calculated the abundance-weighted mean trait value (CWM; Garnier et al. 2004) and FDis (Laliberté and Legendre 2010) for each subplot. The CWM value of the *i*th subplot was calculated separately for each trait *t* as follows:

$${\text{CWM}}_{i}=\sum_{j=1}^{S}p_{ij}t_{j}$$

where *t*_*j*_ is the trait value of species *j, p*_*ij*_ is the relative abundance of species *j* in subplot *i*, and *S* is the total number of species in the forest plot. FDis was based on the abundance-weighted mean trait value of all five functional trait dimensions (Figure S1), which together define a central point (centroid) in functional space. FDis is defined as the mean distance of individual species to the centroid (Laliberté and Legendre 2010; de Bello et al. 2021b). FDis showed low values if all species in the subplot showed similar trait values, for example, through abiotic or biotic filtering, whereas FDis showed high values if the species in the subplot had a greater dispersion in the trait space. Because some traits were highly skewed, the CWM of LA, SLA, and LThick was log-transformed to normalize their distributions.

### 2.2 Environmental Data

Environmental variables were determined within 20 m × 20 m quadrats and divided into three groups: soil nutrients, variables describing canopy structure and variation, and topography-derived variables. These variables are likely to cover all the major aspects of the local environment of the plot. Initially, we considered 24 environmental variables consisting of four topographic variables, 17 soil nutrients, two canopy cover variables, and solar radiation, but removed highly correlated variables (TEB, BS, Res.Inor.N, Al.sat, Mg, Hill shading, pH.H_2_O, Ca, K, and Al). The retained nutrient variables included the three elements Fe, Mn, and Na, extractable phosphorus (Bray.P), effective cation exchange capacity (ECEC), ammonium (NH_4_^+^), nitrate (NO_3_^−^), and phosphate (PO^4^), which were measured using the resin bag method. Variables describing canopy structure and its variation included the mean canopy height of those 1-m^2^ quadrats that showed a high above the median of the quadrat (P50), where high values indicated a subplot with taller trees. We used the coefficient of variation of the top canopy height at 5-m horizontal intervals (CV5m) as a measure of canopy structure heterogeneity. Finally, the topography-derived variables included slope, aspect (sin-transformed), topographic wetness index (TWI), and amount of incoming solar radiation (solar) (see Appendix S2 for further details and Figure S2 for the maps of the variables). In our plot, these variables were heterogeneous and formed patches, particularly soil nutrients, which can be categorized into Ultisol and Oxisol (Brockelman et al. 2017).

### 2.3 Construction of Hierarchical Candidate Models

We developed two candidate models to assess the impact of environmental factors and spatial structure on CWM and FDis. The first candidate model included all environmental variables (Model E). The environmental variables were standardized to have a mean of zero and a standard deviation of one. To address potential multicollinearity among the variables, we conducted a variance inflation factor (VIF) analysis, which resulted in the exclusion of 10 of the 24 variables with high VIF scores (VIF > 3) from further analysis. The influence of space on the distribution of CWM and FDis was then analyzed by adding a continuous spatial field as a random effect to candidate model E, resulting in the creation of a spatial candidate model (Model SE).

### 2.4 Model Framework and Fitting

We developed generalized linear models (GLMs) for the CWM and FDis under the assumption of a Gaussian error structure. Following our a priori candidate models (Models E and SE), we developed two Bayesian GLMs for CWM and FDis. The structure of our most complex model (SE) can be expressed as (Zuur et al. 2017):

$$\begin{array}{c} CFA{\left(\;\text{community}\;\text{functional}\;\text{attribute}\right)}_{i}\sim N(\mu_{i},\sigma^{2})\\ \begin{array}{c} E(CFA_{i})=\mu_{i}\;\text{and}\;\mathrm{var}(CFA_{i})=\sigma^{2}\\ \mu_{i}=\alpha +\beta_{1}(X_{1i})+\beta_{2}(X_{2i})+\ldots +\beta_{\text{n}}(X_{\text{n}i})+u_{i}\\ u_{i}\sim GMRF(0,\text{Σ}) \end{array} \end{array}$$

where *CFA*_*i*_ is the value of a functional attribute (CWM or FDis) at the 20-m subplot *i*, and *X*_n*i*_ is the value of the *n*th environmental variable of subplot *i*. Thus, we assume that the *CFA*_*i*_ values are normally distributed with mean *μ*_*i*_ and variance *σ*^2^. The mean *μ*_*i*_ was modeled as a function of the environmental variables *X*_n*i*_, intercept *α*, and term *u*_*i*_, which represents a continuous spatial field as a random effect. Finally, *u*_*i*_ (i.e., *f*(Space) in Table 1) is a random intercept assumed to be spatially correlated following a Gaussian Markov random field (GMRF) with a mean of 0 and a covariance matrix Σ.

We used the integrated nested Laplace approximation (INLA) modeling approach (Rue et al. 2009) to fit the candidate models. The INLA uses Bayesian inference with a latent Gaussian model to account for spatial autocorrelation. To quantify the spatial relationships (i.e., spatial autocorrelation) among the functional attribute values in each 20-m subplot, a GMRF (a spatial random effect; see Figure S3) was created, where the values of the covariance matrix Σ for the subplots were defined using the Matérn correlation function. The Kappa parameter of the Matérn correlation function, estimated by model fitting, allows us to assess the distance (i.e., range *r*) at which the subplots show spatial dependency (if the Matérn correlation becomes smaller than 0.1). The smaller the kappa value, the larger the distance of the spatial dependency. This approach uses a stochastic partial differential equation (SPDE), which makes the computation of spatial modeling on GMRFs much easier (Lindgren and Rue 2015).

We used INLA non-informative default priors for all regression coefficients of the environmental variables. However, for the spatial effect, we specified a weakly informative prior to minimize spatial confounding (Fuglstad et al. 2019). Spatial confounding, which refers to the collinearity between spatial random effects and covariates in a model (e.g., Marques et al. 2022; Urdangarin et al. 2023), is an important concept in spatial regression modeling. This collinearity can lead to biased estimates of the fixed effects. To account for possible spatial confounding in our study, we employed a “penalized complexity” (PC) prior for the spatial range *r* and marginal standard deviation σ of our spatial effect (Simpson et al. 2017). In this PC framework, the prior shrinks toward an “infinite range and zero variance,” leading to a base model with no spatial effect (Bakka et al. 2018) (reflecting model E). In this context, the shrinkage rate of the prior was specified by selecting hyperparameters with properties *P*(*r* < *r*_0_) = 5% and *P*(*σ* > *σ*_0_) = 5% (e.g., Beguin et al. 2017; Wakefield et al. 2019). We set *r*_0_ to half the size of our study plot (300 m) for all community functional attributes that targeted coarse-scale spatial variations, allowing for small-scale spatial variations in trait data captured primarily by environmental variables. We selected *σ*_0_ as the 5% quantile of standard deviation expectations for each community functional attribute. For each attribute, we ran boot-strap resampling and obtained 1000 sample standard deviations and then fixed *σ*_0_ at the 5% quantile.

### 2.5 Model Selection and Evaluation

We compared the support for competing candidate models using the deviance information criterion (DIC) and the Watanabe–Akaike information criterion (WAIC). For each community functional attribute, the candidate model with the lowest DIC and WAIC scores was considered most parsimonious. When two or more models performed equally (i.e., ΔDIC < 3), we selected the model with the lowest number of explanatory variables. We also visualized the distribution of posterior probabilities to replicate the original observations using this model. For a well-calibrated model, posterior probabilities are uniformly distributed (Lucas et al. 2020). To assess predictive performance, we used the conditional predictive ordinates (CPO) provided by INLA, which correspond to the leave-one-out predictive density for each observation. The sum of the log-CPO values yields the log pseudo marginal likelihood (LPML), where higher values indicate better predictive ability. We also recorded the number of CPO failures, which reflect numerical instabilities during computation, with values close to zero indicating reliable estimates. We also assessed residual spatial autocorrelation using Moran’s I with distance-based spatial weights (Moran 1950).

### 2.6 Null Model Analysis of Species Turnover Effects

We implemented a null model framework to find out whether the contribution of environmental factors suggested by our statistical models might arise from the observed species abundances alone even for randomly assigned traits. To this end, the “trait shuffle” null model procedure (Duarte et al. 2018) involved randomly permuting the values of species traits among species, while preserving community composition and species abundances within each 20 m × 20 m subplot. This procedure maintains the observed spatial turnover of species along environmental gradients but removes any direct association between the focal species trait and environmental variables. For each permutation, CWM trait values were recalculated, and the relationship between CWM trait and environmental variables was estimated. Observed slopes between environmental variables and CWM were then compared with null distributions generated from 999 permutations to evaluate whether trait–environment relationships exceeded expectations from the observed species abundances alone. Further details of the null model procedure are provided in Appendix S4.

All analyses were performed using the R statistical software (R Core Team 2022). PCA of the trait data was conducted using the FactoMineR package (Husson et al. 2025). FDis and CWM were calculated using the *dbFD* function in the *FD* package (Laliberté et al. 2022) and a modification of it (dbFD_parallel function; Kim 2018), respectively. INLA analysis and model evaluation were performed using *R-INLA* (Lindgren and Rue 2015) and *INLAutils* (Lucas et al. 2020) packages, respectively. Bootstrap resampling was performed using the *boot* package (Canty and Ripley 2024).

## 3 Results

### 3.1 Model Comparison and Selection for Community Functional Traits

We modeled the CWM of each trait separately and the FDis for each of the five trait dimensions individually and for all traits together. The spatial model performed in all cases significantly better than the non-spatial model according to consistent DIC and WAIC values (Table 1). Between the models, spatial models showed higher LPML, with no CPO failures, indicating better predictive performance compared to non-spatial models (Table S1). Our results suggest that spatial patterns in community functional attributes are influenced by both abiotic factors due to environmental gradients and spatially structured processes. These spatial patterns highlight the interconnectedness of plant communities and their abiotic and biotic environments, emphasizing the importance of spatial considerations in trait–environment relationship studies.

### 3.2 Effect of Environmental Variables on Community Functional Attributes

The effects of environmental variables on variations in the community functional attributes are shown in Table 2. In the final selected models, which always included a spatial field, the relationships between community functional attributes and environmental variables were both positive and negative, and all functional attributes showed at least one significant association with the environmental variables. The local variation in the community-weighted mean tree height (CWM.TH) was significantly related to four environmental variables, including the TWI, mean canopy height above the median (P50), variation of the top canopy height (CV5m), and Fe, and leaf dry matter content (CWM.LDMC) was related to the same variables, except P50. The remaining traits were related to one or two environmental variables each (Table 2).

The TWI was the explanatory variable with the most significant effect on the variation in the CWM of the functional traits (Table 2). TWI had a positive effect on SLA, suggesting that water availability favors species with high photosynthetic rates. It showed negative effects on LThick, LDMC, WD, and TH, suggesting a conservative resource-use strategy for plants in the dry areas of the study plot. TWI was followed by canopy structure variables, mean canopy height above the median (P50), variation of the top canopy height (CV5m), and soil nutrient Fe. P50 showed positive relationships with leaf area, leaf greenness (SPAD), and TH, whereas CV5m and Fe showed negative and positive relationships with LDMC and TH, respectively. Solar radiation positively correlated with leaf thickness. Mn showed a negative relationship with WD. The remaining variables did not have a significant effect on the CWM of different traits.

**Figure 2:**
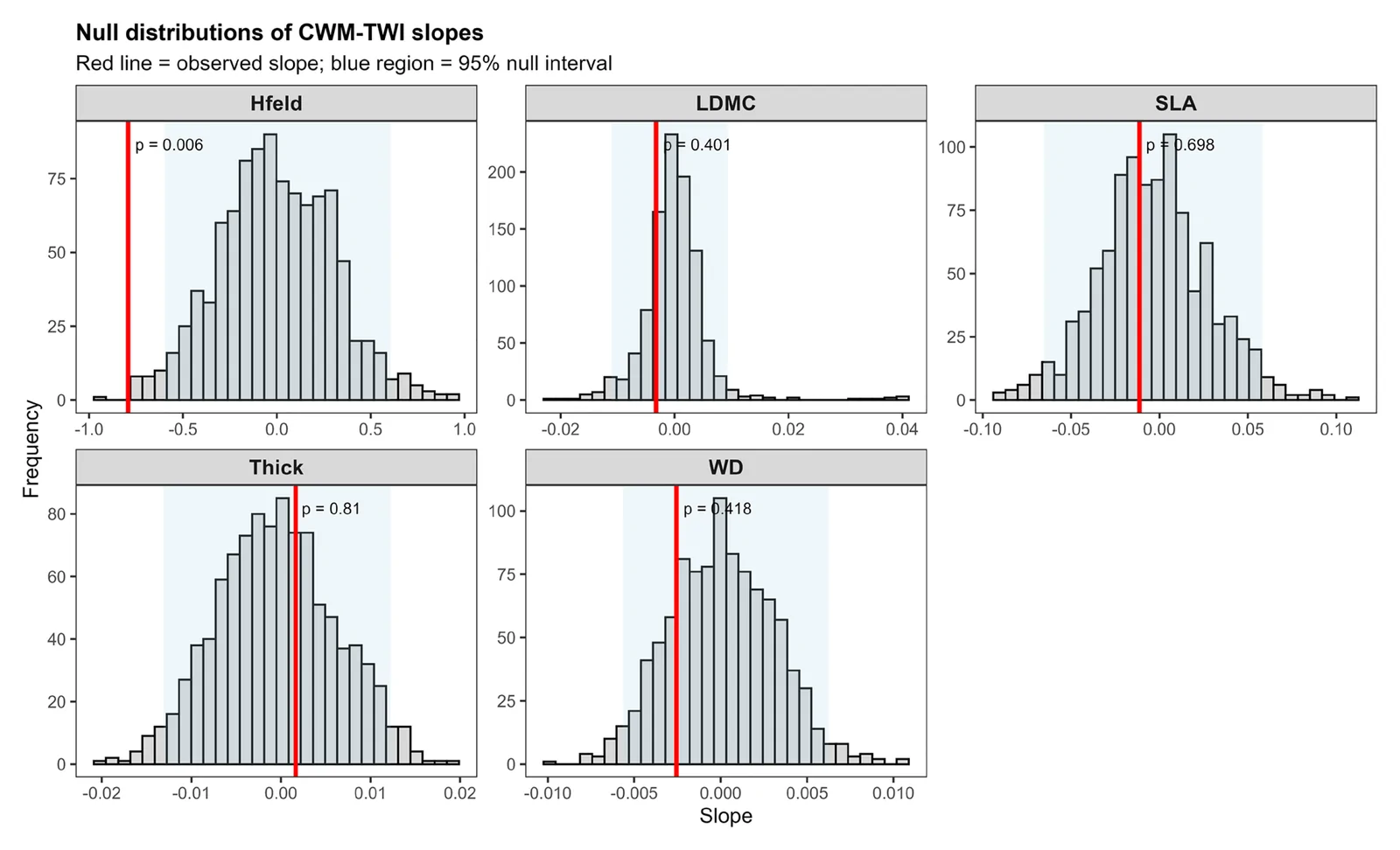
Null distributions of trait–environment slopes under environmentally structured species turnover for TWI, identified as a major driver in the spatial SE model (see Table 1). For each trait, histograms show the null distributions of slopes obtained by permuting species trait values among species while maintaining community composition unchanged. The vertical red line indicates the observed slope between the trait and a given trait relationship. If the observed slope falls within the null distribution, the trait–environment association is consistent with species turnover along the environmental gradient. If the observed slope falls outside the null distribution, the relationship indicates environmentally structured trait variation beyond species turnover. In our analysis, all traits except tree height fall within the null expectations. Hfeld: Tree height, Thick: Leaf thickness. See Appendix S4 for further details on the null model procedure and Figure S8 for null model results for the remaining important variables.

Null model results further showed that, for most traits, the observed slopes between CWM and environmental variables fell within the null expectations, suggesting that the observed trait–environment relationships were driven by species turn-over along different environmental gradients. In contrast, TH showed a deviation from null expectations, suggesting environmentally structured trait variation beyond species turnover (Table S2; Figures 2 and S8).

The FDis of the different trait dimensions changed within our 30-ha plot in response to several environmental variables, and the spatial models showed effects similar to those of the CWM (Table 2). However, the mean canopy height above the median (P50), which distinguishes gaps from mature forest, was the explanatory variable with the most significant effect on variation in trait dispersion. The dispersion of the *light-acquisitive* dimension (FDis_ACQ_) increased with the mean canopy height above the median (P50) and residual ammonium in the soil (Res.NH_4_^+^), which is the amount of ammonium available for plant uptake as an inorganic nitrogen source. Dispersion of the *leaf structural* dimensions (FDis_STR_) increased with the TWI and slope, whereas dispersion of leaf greenness (FDis_SPAD_) decreased with slope and variation of the top canopy height (CV5m) and increased with P50 and Fe. The dispersion of wood density (FDis_WD_) decreased with increasing TWI and Mn content. The dispersion of tree height (FDis_TH_) increased with P50 and Mn, and decreased with solar radiation and Fe levels. The dispersion of all traits (FDis_ALL_) increased with increasing P50 values.

### 3.3 Effect of Spatial Structure

The incorporation of a spatial field greatly improved the fit of the models, with differences in the evaluation metric scores between the spatial and non-spatial models ranging from 180 to 950 (a difference score of zero defined as the best model; Table 1). Maps of the estimated spatial random effects and predictions of the selected models for different community functional attributes are shown in Figure S5. The spatial patterns of the estimated random effects were closely associated with the observed spatial patterns of community functional attributes (Figure 1), indicating that a substantial proportion of variation in community functional composition is spatially structured, which is not captured by the environmental variables. This suggests that spatially structured processes contribute to the observed patterns, whereas the effects of the measured environmental variables are comparatively small.

The inclusion of a spatial field strongly affected the significance of the relationships between functional traits and environmental variables. In the non-spatial models (Model E in Figure S4), functional traits showed significant relationships with six (out of 14) environmental variables. However, accounting for spatially structured processes in these models reduces the number of significant relationships to an average of only two variables. For the CWM of LDMC and, to some extent, for TH, space had a weak effect as these variables retained half of their significant relationships when including a spatial field. Both selected similar explanatory variables (Figure S4).

## 4 Discussion

Understanding the mechanisms underlying plant community assembly requires disentangling the roles of environmental variation, spatial structure, and species composition in shaping community functional patterns. In this study, we combined spatially explicit modeling with null model tests to evaluate these components in a species-rich tropical forest. Our results show that a large proportion of variation in community functional composition and diversity is spatially structured, as captured by the spatial component in the INLA analysis that is unrelated to the environmental variables used in the analysis. Considering that our analysis was carried out based on a fine-grained dataset, it is not surprising that the spatial signal was high. It shows that plant distribution and community functional composition is not only a result of environmental gradients, but also other spatially structured processes contribute to the observed patterns. After accounting for spatial structure, most of the important trait–environment relationships identified by the INLA analysis could be explained by environmentally structured species turnover (i.e., the component of variation in species composition aligned with environmental gradients), with low evidence for additional trait–environment associations beyond species compositional change. Importantly, the null model retains the observed species composition, including the effects of biotic and spatial processes, and tests whether composition’s alignment with environmental gradients alone is sufficient to explain the observed trait–environment relationships.

Our results suggest that CWM trait patterns at our plot (20 m × 20 m resolution within a 30-ha plot) are primarily explained by species composition and spatially structured processes, and that the observed trait–environment relationships largely arise through environmentally structured species turn-over rather than direct trait-level responses to environmental gradients. Detecting the direct trait-level responses likely requires broader environmental gradients or the inclusion of intraspecific trait variation. Nevertheless, species composition and resulting turnover are likely linked to environmental variation, as previous work in this forest identified species assemblages associated with soil nutrients and other habitat features (Chanthorn et al. 2024).

Among the environmental variables considered, topographic water availability (TWI) emerged as the primary gradient associated with community functional composition and diversity. There was only limited evidence for a direct association beyond species turnover (with the exception of TH). Other variables, including soil nutrients and canopy structure, showed weaker or nonsignificant effects, once spatial structure was accounted for. This suggests that local hydrology plays a key role in shaping community functional composition and diversity in this tropical plot, which is consistent with plants’ acquisitive growth in wetter microsites and more conservative resource use in drier areas strategies.

Our analyses also highlight the importance of accounting for spatial autocorrelation when evaluating trait–environment relationships. Ignoring spatial structure led to inflated significance of environmental variables, whereas explicitly incorporating a spatial field allowed us to isolate more robust environmental signals. This confirms that spatial non-independence can obscure the interpretation of trait–environment relationships and under-scores the need to jointly consider spatial structure and species composition when inferring community assembly processes.

### 4.1 Trait–Environment Relationships Through Species Turnover Versus Environmentally Structured Trait Variation

Community ecology emphasizes that trait–environment relationships observed in ecological communities can emerge through multiple assembly processes (Kraft et al. 2008; Lepš et al. 2011; Swenson et al. 2012; Peres-Neto et al. 2017; Zelený 2018). In essence, environmental gradients may filter species possessing particular functional traits, whereas competitive interactions and niche differentiation can further structure trait distributions within communities. Consequently, community-level trait patterns may reflect both direct trait-level responses to environmental gradients and the observed species composition along environmental gradients (Kraft et al. 2008).

In this study, we explicitly separated these mechanisms by combining spatially explicit environmental models with null model tests that disentangled trait–environment relationships into components driven by environmentally structured species turn-over versus additional trait–environment associations beyond compositional change.

Our spatially explicit SE models (INLA-SPDE) provided estimates of environmental effects on community functional composition and diversity while explicitly accounting for spatial autocorrelation. This was necessary to obtain unbiased estimates of these environmental effects, as residual spatial auto-correlation can inflate or obscure environmental relationships in non-spatial models, as seen in the E models. The subsequent null model analysis randomized trait values among species while retaining the observed species composition, thereby preserving all underlying ecological processes shaping community structure, including biotic interactions and dispersal processes. This allowed us to evaluate whether the observed environmental relationships could be explained by species composition alone.

### 4.2 The Relationship Between Environmental Variables and Trait Distributions

The TWI emerged as the primary environmental factor associated with spatial variation in community functional traits and these relationships are largely explained by environmentally structured species turnover, indicating that variation in species composition aligned with the environmental gradient drives the observed patterns after accounting for spatial autocorrelation. Topographically controlled hydrology is likely a major factor shaping tree species distributions and functional community composition in the Mo Singto plot, which is characterized by a small seasonal stream running through the middle and low-lying valley areas (Brockelman et al. 2017). Although TWI does not measure actual water availability directly, it provides a reliable proxy for fine-scale soil moisture patterns, which may play a crucial role in structuring local plant communities (Sørensen et al. 2006; Araya et al. 2011; Oddershede et al. 2015).

The restricted range of high values of SLA in the plot points to habitat filtering driven primarily through species compositional turnover (Figures 1A and S8). The positive SLA–TWI relationship suggests that wetter areas are dominated by species with high SLA, indicative of fast growth and rapid biomass accumulation (Poorter and Bongers 2006; Katabuchi et al. 2012; Reich 2014). In contrast, low SLA values reflect a more conservative resource-use strategy by species (Reich et al. 2003), indicating that species in relatively dry areas of the plot use resources conservatively. Notably, TH is an exception, showing a functional signal beyond species composition alone that might indicate environmental filtering (Figure S8). Linking SLA or its inverse, leaf mass per area, to photosynthetic capacity requires careful interpretation, which is context-dependent based on leaf life span variation (Funk and Cornwell 2013; Osnas et al. 2018).

WD similarly reflected compositional turnover along the TWI gradient: Higher WD values, typical characteristics of late-successional species, were concentrated in drier areas where denser wood provides greater mechanical support to species (Preston et al. 2006; Fortunel et al. 2014). Leaf thickness patterns further showed this compositional structuring along the TWI gradient. Thicker leaves occurred predominantly in low-TWI areas and high solar radiance, consistent with species adapted to water-limited environments (Witkowski and Lamont 1991; Niinemets 2001; Katabuchi et al. 2012). Positive correlations with solar radiation also align with expectations that in high light-available environments, plants can produce leaves with enhanced photosynthetic potential without being too costly in terms of resource use for the construction of a high leaf surface area (Niinemets 2001). TH exhibits a negative relationship with TWI, indicating that trees tend to be smaller in the lower, wetter areas—a growth pattern that deviates from what is expected under species turnover mechanism alone (Figure S8), highlighting the importance of considering both species composition and environmental filtering effects. Local canopy structure, variation in evapotranspiration, and permanent gaps covered by lianas along the stream likely contribute to this TH pattern (Salas-Morales et al. 2018; Chanthorn et al. 2024).

Among all traits, LDMC and TH were related to the soil nutrient, Fe. Our results align with Hodgson et al. (2011), who advocated using LDMC as a reliable indicator of gradients in soil fertility independent of shade.

### 4.3 The Spatial Effect in Traits

Our analysis, based on the INLA-SPDE approach, allows us to estimate and account for the effects of space using spatial covariance in modeling trait–environment relationships. This method captures spatial autocorrelation, which serves as a proxy for unmeasured biotic influences and reflects their cumulative effects on trait distributions. Although our study did not directly measure spatially structured biotic processes such as species interactions, dispersal limitations, and local adaptation processes, the spatial components in our models likely encapsulate these effects. This approach aligns with the growing recognition in ecological research that spatial patterns in species distributions and trait variations are not solely determined by environmental factors but are also shaped by processes such as dispersal, biotic interactions, and historical processes (Dirnböck and Dullinger 2004). Furthermore, the spatial component in our models may capture the effects of unmeasured environmental variables or historical factors that influence the current trait distributions. This highlights the complex nature of trait–environment relationships and the importance of considering both abiotic and biotic factors in ecological research.

The estimated spatial effects of traits and large differences in evaluation scores between the non-spatial and spatial models (Table 1) indicate that community functional traits show a strong spatial structure that must be considered when evaluating the effects of environmental variables. Misidentification of significant relationships with environmental variables due to strong spatial structure occurred in all environmental variable groups, but the effect was particularly large for soil nutrients. Many studies in tropical regions have reported that soil properties play an important role in the structure and function of plant communities. However, soil nutrients exhibit strong spatial patterns and significant spatial autocorrelations (John et al. 2007). Pseudoreplication due to spatial autocorrelation is a common problem associated with ecological data, and ignoring it will increase Type I error rates (Legendre 1993). By incorporating spatial autocorrelation using the INLA-SPDE approach, our study contributes to a more comprehensive understanding of the factors shaping plant trait distributions.

Interestingly, our spatial models identified several relationships that were not detected by non-spatial models (Figure S4), including topography, forest canopy structure, and solar radiation (Liu et al. 2014; Oddershede et al. 2015; Pescador et al. 2021). The strong spatial structure of soil nutrients may obscure the effects of these variables by incorrectly capturing the variations in functional traits. The incorporation of a spatial field revealed the spatial structure of functional traits that were independent of the measured environmental factors, thereby revealing the true nature of the trait–environment relationships.

We note that our use of PC priors for the spatial field helps mitigate the issues of spatial confounding, where the effect of a spatially structured environmental driver could be erroneously attributed to spatial random effects (Simpson et al. 2017). Our approach conservatively incorporates only spatial effects that explain the variance beyond that captured by the fixed covariates. Spatial confounding is often overlooked in practice because of the lack of a clear, “unique general definition” and “a definitive solution” (Urdangarin et al. 2023). A potential future direction for our study is to explore the presence of spatial confounding in spatial models (e.g., Marques et al. 2022). This exploration could further enhance our understanding of the spatial effects of trait– environment relationships.

## 5 Conclusions

Our study provides a spatially explicit assessment of community assembly processes and advances our understanding of how environmental gradients and spatial structure shape community functional composition of plant communities. Topographic water availability emerged as the primary environmental gradient associated with patterns in community functional composition and diversity in our plot. This hydrological gradient aligns functional composition along the core axis of tropical plant strategy trade-offs, with acquisitive growth strategies dominating wetter microsites and conservative resource-use strategies prevailing in drier areas.

At the same time, strong spatial autocorrelation in trait distributions substantially influences the perceived strength of environmental effects. By applying the INLA-SPDE framework, we accounted for this spatial structure and identified more robust environmental signals in trait–environment relationships. Analyses that ignore spatial dependence can therefore overestimate or misinterpret the role of environmental drivers.

Using null model tests on these spatially corrected relationships, we further show that most trait–environment associations are consistent with environmentally structured species turnover, whereas certain traits (TH in our case) exhibit additional trait– environment associations beyond species compositional change. This indicates that trait–environment patterns in tropical forests can arise through multiple processes and that spatially explicit models combined with null model inference provide a useful framework for distinguishing these mechanisms. More broadly, our results highlight that interpreting trait–environment relationships requires jointly accounting for spatial structure and species turnover, as community functional patterns may emerge through multiple assembly processes along environmental gradients.

## Supplementary Material

Appendices

## Figures and Tables

**Table 1:** Changes in evaluation metric scores among different generalized linear models applied to community functional attributes, specifically community-weighted mean (CWM) and functional dispersion (FDis), in an old-growth tropical forest in Thailand.

Community functional attributes	ΔDIC				ΔWAIC					Moran’s I					
	Non-spatial		Spatial		Non-spatial		Spatial		Non-spatial				Spatial		
	Env(E)		Env + *f*(Space) (SE)		Env (E)		Env + *f*(Space) (SE)		Estimate		*p*		Estimate	*p*	
(A) Leaf acquisitive															
CWM.LA	178.6		0		170.4		0		0.232		< 0.001		−0.0991	1	
CWM.SLA	371.2		0		363.3		0		0.351		< 0.001		−0.1613	1	
FDiS_ACQ_	254.5		0		241.5		0		0.274		< 0.001		−0.1486	1	
(B) Leaf structure															
CWM.LThick	360.6		0		350.9		0		0.349		< 0.001		−0.1522	1	
CWM.LTough	681.8		0		677		0		0.498		< 0.001		−0.1643	1	
CWM.LDMC	315.5				305.4				0.297		< 0.001		−0.1642	1	
FDis_STR_	482.8		0		477.9		0		0.365		< 0.001		−0.1717	1	
(C) SPAD															
CWM.SPAD	548.9		0		539.5		0		0.458		< 0.001		−0.1536	1	
FDis_SPAD_	566.6		0		555.9		0		0.470		< 0.001		−0.1358	1	
(D) WD															
CWM.WD	556		0		547.4		0		0.470		< 0.001		−0.1562	1	
FDis_WD_	955.9		0		963.1		0		0.559		< 0.001		−0.2135	1	
(E) Tree height															
CWM.TH	744.3		0		749.2		0		0.495		< 0.001		−0.2208	1	
FDis_TH_	384.5		0		372.7		0		0.359		< 0.001		−0.1491	1	
(F) FDis_ALL_	498.3		0		492.1		0		0.393		< 0.001		−0.1617	1	

*Note:* The spatial models incorporated a spatial field to address the spatial correlations in the attributes. In cases where multiple models showed similar performances, we opted for the simplest model with the fewest explanatory variables. ΔDIC = DIC − min(DIC) and the same calculation applies to ΔWAIC. Moran’s I and associated p values assess positive spatial autocorrelation in model residuals. Abbreviations: E, environmental model; SE, spatial environmental model.

**Table 2:** Mean posterior marginal probabilities of fixed effects of environmental variables estimated by the best-performing generalized linear models (Table 1) for community functional attributes, community-weighted mean (CWM) and functional dispersion (FDis).

		Slope	TWI	P50	CV5m	Solar	Res.NH4	Fe	Mn
Leaf acquisitive	CWM.LA			0.034					
	CWM.SLA		0.031						
Leaf structure	CWM.LThick		−0.005			0.006			
	CWM.LTough								
	CWM.LDMC		−0.004		−0.002			0.004	
SPAD	CWM.SPAD			0.059					
WD	CWM.WD		−0.003						−0.001
Tree height	CWM.TH		−0.551	0.249	−0.329			0.912	
Leaf acquisitive	FDis_ACQ_			0.017			0.068		
Leaf structure	FDis_STR_	0.021	0.014						
SPAD	FDis_SPAD_	−0.009		0.007	−0.006	0.014			
WD	FDis_WD_		−0.013						−0.022
Tree height	FDis_TH_			0.007		−0.013		−0.016	0.012
ALL	FDis_ALL_			0.016					

*Note:* A higher value indicates that the variable has a stronger effect than one with a lower value. Colors refer to positive (blue) or negative (rose) effects of the covariates. We show only environmental variables with at least one significant effect on functional attributes. For all mean values, including their credible intervals, see Figure S4. The environmental variables were as follows: Slope, TWI; topographic wetness index, P50; mean canopy height above the median; CV5m, coefficient of variation of the top canopy height at 5-m resolution; solar, solar radiation; Res.NH4, ammonium; Fe, iron; and Mn, manganese. Abbreviations: LA, leaf area; LDMS, leaf dry matter content; LThick, leaf thickness; LTough, leaf toughness; SLA, specific leaf area; SPAD, leaf greenness; TH, maximal tree height; WD, wood density.

## Data Availability

Data and R code for the analyses presented are available via Figshare: https://figshare.com/s/78b8b1ef1e48a20156ff.

## References

[R1] Aguirre-Gutiérrez J, Berenguer E, Oliveras Menor I (2022). Functional Susceptibility of Tropical Forests to Climate Change. Nature Ecology & Evolution.

[R2] Araya YN, Silvertown J, Gowing DJ, McConway KJ, Peter Linder H, Midgley G (2011). A Fundamental, Eco-Hydrological Basis for Niche Segregation in Plant Communities. New Phytologist.

[R3] Bakka H, Rue H, Fuglstad G (2018). Spatial Modeling With R-INLA: A Review. WIREs Computational Statistics.

[R4] Balvanera P, Quijas S, Pérez-Jiménez A (2011). Distribution Patterns of Tropical Dry Forest Trees Along a Mesoscale Water Availability Gradient: Tree Distribution Along Water Availability Gradient. Biotropica.

[R5] Beguin J, Fuglstad G-A, Mansuy N, Paré D (2017). Predicting Soil Properties in the Canadian Boreal Forest With Limited Data: Comparison of Spatial and Non-Spatial Statistical Approaches. Geoderma.

[R6] Bialic-Murphy L, McElderry RM, Esquivel-Muelbert A (2024). The Pace of Life for Forest Trees. Science.

[R7] Borcard D, Legendre P, Drapeau P (1992). Partialling Out the Spatial Component of Ecological Variation. Ecology.

[R8] Brockelman WY, Nathalang A, Maxwell JF (2017). Mo Singto Forest Dynamics Plot: Flora and Ecology.

[R9] Canty A, Ripley BD (2024). boot: Bootstrap R (S-Plus) Functions.

[R10] Cardinale BJ, Duffy JE, Gonzalez A (2012). Biodiversity Loss and Its Impact on Humanity. Nature.

[R11] Chanthorn W, Wiegand T, Nathalang A (2024). Species Assemblages and Their Drivers Differ Between Trees and Lianas in a Seasonal Evergreen Forest in Thailand. Ecosphere.

[R12] de Bello F, Carmona CP, Dias ATC, Götzenberger L, Moretti M, Berg MP (2021a). Handbook of Trait-Based Ecology: From Theory to R Tools.

[R13] de Bello F, Carmona CP, Dias ATC, Götzenberger L, Moretti M, Berg MP (2021b). Community Metrics. Handbook of Trait-Based Ecology: From Theory to R Tools.

[R14] Diniz-Filho JAF, Siqueira T, Padial AA, Rangel TF, Landeiro VL, Bini LM (2012). Spatial Autocorrelation Analysis Allows Disentangling the Balance Between Neutral and Niche Processes in Metacommunities. Oikos.

[R15] Dirnböck T, Dullinger S (2004). Habitat Distribution Models, Spatial Autocorrelation, Functional Traits and Dispersal Capacity of Alpine Plant Species. Journal of Vegetation Science.

[R16] Díaz S, Cabido M (2001). Vive la Différence: Plant Functional Diversity Matters to Ecosystem Processes. Trends in Ecology & Evolution.

[R17] Duarte LDS, Debastiani VJ, Carlucci MB, Diniz-Filho JAF (2018). Analyzing Community-Weighted Trait Means Across Environmental Gradients: Should Phylogeny Stay or Should It Go?. Ecology.

[R18] Fortunel C, Ruelle J, Beauchêne J, Fine PVA, Baraloto C (2014). Wood Specific Gravity and Anatomy of Branches and Roots in 113 Amazonian Rainforest Tree Species Across Environmental Gradients. New Phytologist.

[R19] Fuglstad G-A, Simpson D, Lindgren F, Rue H (2019). Constructing Priors That Penalize the Complexity of Gaussian Random Fields. Journal of the American Statistical Association.

[R20] Funk JL, Cornwell WK (2013). Leaf Traits Within Communities: Context May Affect the Mapping of Traits to Function. Ecology.

[R21] Garnier E, Cortez J, Billès G (2004). Plant Functional Markers Capture Ecosystem Properties During Secondary Succession. Ecology.

[R22] Hodgson JG, Montserrat-Martí G, Charles M (2011). Is Leaf Dry Matter Content a Better Predictor of Soil Fertility Than Specific Leaf Area?. Annals of Botany.

[R23] Hubbell SP (2001). The Unified Neutral Theory of Biodiversity and Biogeography (MPB-32).

[R24] Husson F, Josse J, Le S, Mazet J (2025). FactoMineR: Multivariate Exploratory Data Analysis and Data Mining.

[R25] John R, Dalling JW, Harms KE (2007). Soil Nutrients Influence Spatial Distributions of Tropical Tree Species. Proceedings of the National Academy of Sciences of the United States of America.

[R26] Katabuchi M, Kurokawa H, Davies SJ, Tan S, Nakashizuka T (2012). Soil Resource Availability Shapes Community Trait Structure in a Species-Rich Dipterocarp Forest. Journal of Ecology.

[R27] Kim S (2018). dbFD_parallel.R 24963 Bytes.

[R28] Kraft NJB, Valencia R, Ackerly DD (2008). Functional Traits and Niche-Based Tree Community Assembly in an Amazonian Forest. Science.

[R29] Kühn I (2007). Incorporating Spatial Autocorrelation May Invert Observed Patterns. Diversity and Distributions.

[R30] Laliberté E, Legendre P (2010). A Distance-Based Framework for Measuring Functional Diversity From Multiple Traits. Ecology.

[R31] Laliberté E, Legendre P, Shipley B (2022). FD: Measuring Functional Diversity (FD) From Multiple Traits, and Other Tools for Functional Ecology.

[R32] Legendre P (1993). Spatial Autocorrelation: Trouble or New Paradigm?. Ecology.

[R33] Lepš J, de Bello F (2023). Differences in Trait–Environment Relationships: Implications for Community Weighted Means Tests. Journal of Ecology.

[R34] Lepš J, de Bello F, Šmilauer P, Doležal J (2011). Community Trait Response to Environment: Disentangling Species Turnover vs Intraspecific Trait Variability Effects. Ecography.

[R35] Lindgren F, Rue H (2015). Bayesian Spatial Modelling With R-INLA. Journal of Statistical Software.

[R36] Liu J, Yunhong T, Slik JWF (2014). Topography Related Habitat Associations of Tree Species Traits, Composition and Diversity in a Chinese Tropical Forest. Forest Ecology and Management.

[R37] Lucas T, Python A, Redding D (2020). Graphical Outputs and Spatial Cross-Validation for the R-INLA Package Using INLAutils.

[R38] Marques I, Kneib T, Klein N (2022). Mitigating Spatial Confounding by Explicitly Correlating Gaussian Random Fields. Environmetrics.

[R39] McFadden IR, Bartlett MK, Wiegand T (2019). Disentangling the Functional Trait Correlates of Spatial Aggregation in Tropical Forest Trees. Ecology.

[R40] Moran PAP (1950). Notes on Continuous Stochastic Phenomena. Biometrika.

[R41] Niinemets Ü (2001). Global-Scale Climatic Controls of Leaf Dry Mass Per Area, Density, and Thickness in Trees and Shrubs. Ecology.

[R42] Oddershede A, Svenning J-C, Damgaard C (2015). Topographically Determined Water Availability Shapes Functional Patterns of Plant Communities Within and Across Habitat Types. Plant Ecology.

[R43] Osnas JLD, Katabuchi M, Kitajima K (2018). Divergent Drivers of Leaf Trait Variation Within Species, Among Species, and Among Functional Groups. Proceedings of the National Academy of Sciences of the United States of America.

[R44] Peres-Neto PR, Dray S, ter Braak CJF (2017). Linking Trait Variation to the Environment: Critical Issues With Community-Weighted Mean Correlation Resolved by the Fourth-Corner Approach. Ecography.

[R45] Pescador DS, de Bello F, López-Angulo J, Valladares F, Escudero A (2021). Spatial Scale Dependence of Ecological Factors That Regulate Functional and Phylogenetic Assembly in a Mediterranean High Mountain Grassland. Frontiers in Ecology and Evolution.

[R46] Pillar VD, Duarte LDS (2010). A Framework for Metacommunity Analysis of Phylogenetic Structure. Ecology Letters.

[R47] Pinho BX, de Melo FPL, Arroyo-Rodríguez V, Pierce S, Lohbeck M, Tabarelli M (2018). Soil-Mediated Filtering Organizes Tree Assemblages in Regenerating Tropical Forests. Journal of Ecology.

[R48] Poorter L, Bongers F (2006). Leaf Traits Are Good Predictors of Plant Performance Across 53 Rain Forest Species. Ecology.

[R49] Possen BJHM, Oksanen E, Rousi M (2011). Adaptability of Birch (*Betula pendula* Roth) and Aspen (*Populus tremula* L.) Genotypes to Different Soil Moisture Conditions. Forest Ecology and Management.

[R50] Pothasin P, Paradis E, Brockelman WY (2022). Seed Size Variation of Trees and Lianas in a Tropical Forest of Southeast Asia: Allometry, Phylogeny, and Seed Trait - Plant Functional Trait Relationships. Frontiers in Plant Science.

[R51] Preston KA, Cornwell WK, DeNoyer JL (2006). Wood Density and Vessel Traits as Distinct Correlates of Ecological Strategy in 51 California Coast Range Angiosperms. New Phytologist.

[R52] Punchi-Manage R, Getzin S, Wiegand T (2013). Effects of Topography on Structuring Local Species Assemblages in a Sri Lankan Mixed Dipterocarp Forest. Journal of Ecology.

[R53] R Core Team (2022). R: A Language and Environment for Statistical Computing.

[R54] Reich PB (2014). The World-Wide ‘Fast-Slow’ Plant Economics Spectrum: A Traits Manifesto. Journal of Ecology.

[R55] Reich PB, Wright IJ, Cavender-Bares J (2003). The Evolution of Plant Functional Variation: Traits, Spectra, and Strategies. International Journal of Plant Sciences.

[R56] Rue H, Martino S, Chopin N (2009). Approximate Bayesian Inference for Latent Gaussian Models by Using Integrated Nested Laplace Approximations. Journal of the Royal Statistical Society, Series B: Statistical Methodology.

[R57] Salas-Morales SH, González EJ, Meave JA (2018). Canopy Height Variation and Environmental Heterogeneity in the Tropical Dry Forests of Coastal Oaxaca, Mexico. Biotropica.

[R58] Salazar Villegas MH, Wiegand T, González-M R, Rodriguez-Buritica S, Qasim M, Csaplovics E (2023). Spatial Facilitation and Competition Regulate Tree Species Assembly in a Tropical Dry Forest. Frontiers in Forests and Global Change.

[R59] Seidler TG, Plotkin JB (2006). Seed Dispersal and Spatial Pattern in Tropical Trees. PLoS Biology.

[R60] Silvertown J, Dodd ME, Gowing DJG, Mountford JO (1999). Hydrologically Defined Niches Reveal a Basis for Species Richness in Plant Communities. Nature.

[R61] Simpson D, Rue H, Riebler A, Martins TG, Sørbye SH (2017). Penalising Model Component Complexity: A Principled, Practical Approach to Constructing Priors. Statistical Science.

[R62] Sørensen R, Zinko U, Seibert J (2006). On the Calculation of the Topographic Wetness Index: Evaluation of Different Methods Based on Field Observations. Hydrology and Earth System Sciences.

[R63] Swenson NG, Erickson DL, Mi X (2012). Phylogenetic and Functional Alpha and Beta Diversity in Temperate and Tropical Tree Communities. Ecology.

[R64] Urdangarin A, Goicoa T, Ugarte MD (2023). Evaluating Recent Methods to Overcome Spatial Confounding. Revista Matemática Complutense.

[R65] Wakefield J, Fuglstad G-A, Riebler A, Godwin J, Wilson K, Clark SJ (2019). Estimating Under-Five Mortality in Space and Time in a Developing World Context. Statistical Methods in Medical Research.

[R66] Wang X, Sun S, Sedio BE (2022). Niche Differentiation Along Multiple Functional-Trait Dimensions Contributes to High Local Diversity of Euphorbiaceae in a Tropical Tree Assemblage. Journal of Ecology.

[R67] Witkowski ETF, Lamont BB (1991). Leaf Specific Mass Confounds Leaf Density and Thickness. Oecologia.

[R68] Wright IJ, Reich PB, Westoby M (2004). The Worldwide Leaf Economics Spectrum. Nature.

[R69] Zelený D (2018). Which Results of the Standard Test for Community-Weighted Mean Approach Are Too Optimistic?. Journal of Vegetation Science.

[R70] Zuur AF, Ieno EN, Saveliev AA (2017). Beginner’s Guide to Spatial, Temporal and Spatial-Temporal Ecological Data Analysis With R-INLA.

