# Supplementary material for: Spatial Regulation of Trait–Environment Interactions Shapes Plant Community Functional Patterns in a Tropical Forest
Source: J Veg Sci. Author manuscript; Available in PMC 2026 Aug 31. (PMC7619427; doi:10.1111/jvs.70169)
Supplement: Supplementary file 1 [file EMS217624-supplement-Appendices.docx]

Online Supplementary Information:

**Data Archiving Statement:** Data and R code for the analyses presented are available via Figshare: <https://figshare.com/s/78b8b1ef1e48a20156ff>

**Spatial regulation of trait–environment interactions shapes plant community functional patterns in a tropical forest**

Rajapandian Kanagaraj, Warren Y. Brockelman, Thorsten Wiegand, Anuttara Nathalang, Jie Yang, Nitin K. Tripathi, Wirong Chanthorn

**Appendix S1: Tree census**

The Mo Singto plot is a member of the ForestGEO network (Davies et al., 2021). Based on the ForestGEO standard protocol, the plot is divided into 20-m ×20-m quadrats and all trees with DBH ≥ 1 cm are tagged, mapped, identified to species and their DBH measured. A re-census is conducted every five years. Here we used the tree data of the census conducted from 2015 to 2017, which are the closest to the period in which environmental variables were collected.

**Appendix S2: Environmental variables**

*Soil nutrients*

Soil sampling followed a combined systematic and random sampling design where 180 sampling locations were located at a regular grid width, and 90 sampling locations were randomly distributed at distances of 2, 8, or 20 m from each second sampling location of the regular grid. This scheme allowed for an extrapolation at the 20-m × 20-m scale by the kriging (John et al., 2007). Soil with litter excluded was sampled at 0–10 cm depth. In total, 250 soil samples were taken during May–June 2017, and they were analyzed for 26 different forms of nutrients and for pH at the soil chemical laboratory of the Smithsonian Research Institute in Panama. These variables comprised eight elements, i.e. N, P, K, Al, Mg, Fe, Ca and Na. The soil analyses are standard analyses that have been applied to many ForestGEO plots across tropical forests (Bunyavejchewin et al., 2019; Davies et al., 2021).

*Forest structure*

Forest structure variables were extracted from a 1-m resolution canopy height model representing the top-of-canopy height (TCH) estimated from airborne LiDAR data acquired in April 2017 (Jha et al., 2020). We extracted several variables from the top-of-canopy height data that allowed us to assess responses to different degrees of disturbance. More specifically, for each 20-m ×20-m quadrat, we calculated the mean canopy height of 1 m^2^ quadrats that were above the 50^th^ percentile of the quadrat, resulting in the variable P50. For example, the corresponding variable P10 would be the mean canopy height above the 10% percentile and therefore close to the mean canopy height (which would be P0). Similarly, P99 would be basically the maximal canopy height. However, the variable P50 does not capture variability of the canopy structure within a quadrat, which is important for determining forest successional stage, particularly at the intermediate or stem exclusion stage with low heterogeneity of canopy structure (Chanthorn et al., 2016). Thus, we computed additionally the coefficient of variation of the top canopy height at 5-m resolution (CV5m) over each 20-m × 20-m quadrat as a measure of heterogeneity of canopy structure.

*Topography*

We used topographic variables similar to those used in previous studies conducted in Panama and Sri Lanka (Kanagaraj et al., 2011; Punchi-Manage et al., 2013). We estimated slope, aspect, and curvature from a 1-m resolution digital elevation model (DEM), which was derived from the airborne LiDAR data (Jha et al., 2020). However, we transformed the raw data of aspect that range between 0 and 360 degrees into values ranging from −1 to 1 using the sine function to accommodate the circular nature of the data. Because some aspect values may have a higher risk of mortality or branch falling due to higher wind exposure, we weighted aspect by adding the average wind direction in a year, i.e., approximately 150 degrees of azimuth. In addition to these variables, two topography-derived variables, the amount of incoming solar radiation (insolation), and the topographic wetness index, were estimated using the SAGA GIS software (http://www.saga-gis.org). We used the solar radiation because we observed that some areas of our plot that receive less radiation are densely covered by understory perennial shrubs such as *Strobilanthes* spp., which may affect tree seedlings (Chanthorn et al., 2013). Solar radiation (insolation) was calculated every two hours during the daytime in the year 2016, accounting for slope, aspect, and atmospheric properties, and finally summed into a yearly value as “kilowatt-hours per m^2^” (Kwh/m^2^). The topographic wetness index indicates the capacity of an area to accumulate soil moisture based on topography. Water flow in a landscape is also evaluated in the calculation of the wetness index. Thus, for estimation of the topographic wetness index we included the surrounding area within 1 km of the plot border as a buffer. Our plot is located on a watershed with two small streams running through it, which create strong contrasts of wetness.

Appendix S3: Functional traits (modified from Pothasin et al., 2022)

*Leaf traits*

The protocol of Pérez-Harguindeguy et al. (2013) was followed and leaf traits were based on samples of at least 32 individual plants of different sizes per species. At least three non-damaged leaves per individual tree were used to measure leaf economic spectrum traits. Sun-exposed leaves were collected with a long pole, whereas trained tree climbers collected liana leaves. Each leaf was scanned and leaf greenness (chlorophyll content) was measured using a portable greenness meter. Leaf thickness and toughness was measured three times at the upper, middle, and lower parts of each leaf blade. Finally, the same leaves were dried at 70°C for 3 days and weighed to obtain specific leaf area (leaf area per mass unit). The area of each individual leaf from the scammed pictures was determined by using the R package “LeafArea” (Katabuchi, 2015). Overall, leaves of 146 tree species and 46 liana species were measured.

*Wood Density and Maximum Tree Size*

Wood density values were derived mainly from the global wood density database (Chave et al., 2009). When wood densities were not available at the species level, genus level and family level (for 43.2 and 17.8% of species, respectively) were used. Maximum tree heights were calculated using the DBH–height allometric equation developed for Southeast Asian tropical forests (Feldpausch et al., 2010). Tree height was calculated with the BIOMASS R package (Réjou‐Méchain et al., 2017).

**Appendix S4: Null model analysis**

We implemented a null model framework in order to assess the influence of environmentally structured species turnover mechanism and environmentally structured trait variation beyond species composition mechanism to the observed trait–environment relationships. The framework evaluated whether the observed relationships came from environmentally structured species turnover mechanism or from environmentally structured trait variation beyond species composition mechanism. To this end, for each trait, we used the community-weighted mean (CWM) trait values, calculated for each 20 m × 20 m subplot using species abundance data and species trait values, and quantified the relationship between the given CWM trait and the environmental variables identified important in the spatial SE model (Table 1) using simple linear regression model (β_obs_).

To generate null expectations, we randomly permuted the species trait values among species while we preserved the community composition and species abundances in each subplot intact. This randomization maintains the observed species turnover across the environmental gradient but removes any direct association between species traits and environmental conditions. For each permutation, we recalculated the CWM values for each 20 m × 20 m and estimated the corresponding slope of CWM trait–environmental variable relationship using simple linear regression model (β_null_). This procedure was repeated 999 times to generate null distributions of regression slopes for each trait.

The observed slopes were then compared to the null distributions using a standardized effect size (SES):

SES = (β_obs_ – mean(β_null_)) / sd(β_null_)

where β_obs_ is the observed slope and β_null_ represents slopes from the permuted datasets for a given trait. Permutation based p-values were calculated as the proportion of null slopes with absolute values equal to or greater than the observed slope.

For a given trait, if the observed slope fell within the null distribution, the trait–environment relationship was interpreted as consistent with the role of environmentally structured species turnover mechanism. Conversely, for traits in which the observed slopes going beyond the null expectations indicate the role of environmentally structured trait variation beyond species compositional turnover mechanism.

**Appendix S5: Extent of spatial dependency in community functional trait distributions**

The distance (range) at which spatial dependency diminished in the community functional attributes ranged between ~80 m for the functional dispersion of *leaf structural* dimension and 170 m for the functional dispersion of the wood density dimension (Fig. S9C), which reflects the patch sizes of the maps of the spatial random effects, as shown in Figure S5.

The spatial field of INLA-SPDE can capture spatial dependency patterns in response data that are not accounted for by measured environmental variables across multiple spatial scales (Paradinas et al., 2022). This can complicate the ecological interpretation of the spatial field as both unmeasured environmental variables and spatially structured processes can act at multiple spatial scales. Hence, some uncertainty remains in the interpretation of spatially structured variations that cannot be explained by environmental variables. However, given that we included a wide range of environmental variables, including topography, soil nutrients, solar radiation, and canopy structure, we assumed that the estimated spatial field is mostly influenced by biotic forces rather than by unmeasured environmental variables. For example, the spatial aggregation of locally dominant species can lead to spatial autocorrelation in community-weighted mean trait values. Spatial aggregation is prevalent in tropical forests and can be caused by dispersal limitations and contagious animal seed dispersal if animals defecate seeds in clumps under fruiting trees, latrines, or resting sites (Chanthorn et al., 2018). The estimated spatial clustering of traits, which was independent of the measured environmental variables, showed different spatial patterns and cluster sizes, suggesting that different mechanisms may be involved in the tree community assembly in our plot. For example, trait distributions can contract as a result of biotic competition that takes place at small spatial scales for resources such as light, water, and nutrients, but facilitation can expand local trait distributions (Butterfield & Callaway, 2013).

**FIGURE S1**. PCA biplot colored by plant trait contribution to significant PCs (High: red; Medium: yellow; Low: blue). The biplot with traits was also labeled by biological group (A: Acquisitive; S: Structural; O: Others). LA, leaf area; SLA, specific leaf area; Thick, leaf thickness (LThick); Tough, leaf toughness (LTough); LDMC, leaf dry matter content; SPAD, leaf greenness; WD, wood density; Hfeld, tree height (TH). Note: LA, SLA and Thick contributed high to the PCs, but indicated trait oppositions in PCA space (LA and SLA vs. Thick), revealing a leaf economic spectrum.

| A) Topographic, canopy cover and solar radiation variables | |
| --- | --- |
|  |  |
|  |  |
| B) Soil nutrient variables |  |
|  |  |
|  |  |
| **FIGURE** **S2**. Spatial variation of environmental variables at 20 m × 20 m scale in the study plot. | |

**FIGURE S3.** The spatial mesh for the study area is used to build the stochastic partial differential equation (SPDE) for the spatial generalized linear models fitted to community functional attributes.

| **A) Leaf acquisitive** | | | **B) Leaf structure** | | | | |  |
| --- | --- | --- | --- | --- | --- | --- | --- | --- |
| CWM.LA | CWM.SLA | FDis_ACQ_ | | CWM.LThick | CWM.LTough | CWM.LDMC | FDis_STR_ | |
|  |  |  | |  |  |  |  | |

**FIGURE S4**. Continued.

| **C) SPAD** | | **D) WD** | | **E) Tree height** |  | **F)** |
| --- | --- | --- | --- | --- | --- | --- |
| CWM.SPAD | FDis_SPAD_ | CWM.WD | FDis_WD_ | CWM.TH | FDis_TH_ | FDis_ALL_ |
|  |  |  |  |  |  |  |

**FIGURE S4.** Plots depicting the posterior marginal probabilities (x-axis) of fixed effects (y-axis) as estimated by the spatial generalized linear models, as detailed in Table 1, for the community functional attributes, specifically the community-weighted mean (CWM) and functional dispersion (FDis). For comparative purposes, parameter estimates for models excluding the SPDE (E: non-spatial GLM incorporating only environmental variables) are illustrated in grey. SE denotes the Spatial GLM with environmental variables.

|  | Community-weighted mean (CWM) | | |  | |  |
| --- | --- | --- | --- | --- | --- | --- |
|  | A) Leaf acquisitive | | | B) Leaf structure | |  |
|  | LA | SLA |  | LThick | LTough | LDMC |
| Spatial effect |  |  |  |  |  |  |
| Prediction |  |  |  |  |  |  |
|  |  |  |  |  |  |  |
|  | C) SPAD | D) WD | E) Tree height |  |  |  |
|  | SPAD | WD | TH |  |  |  |
| Spatial effect |  |  |  |  |  |  |
| Prediction |  |  |  |  |  |  |
|  |  |  |  |  |  |  |
|  | Functional dispersion (FDis) | |  | |  |  |
|  | FDis_ACQ_ | FDis_STR_ | FDis_SPAD_ | FDis_WD_ | FDis_TH_ | FDis_ALL_ |
| Spatial effect |  |  |  |  |  |  |
| Prediction |  |  |  |  |  |  |
|  | **FIGURE S5.** Estimated spatial random effects and predictions of community functional attributes, specifically the community-weighted mean (CWM) and functional dispersion (FDis) by the best performing GLMs (see Table 1). | | | | | |

| **A) Leaf acquisitive** | |
| --- | --- |
| Log(CWM.LA) | Log(CWM.SLA) |
|  |  |

| FDis_ACQ_ |  |
| --- | --- |
|  |  |

| **B) Leaf structure** | |
| --- | --- |
| Log(CWM.LThick) | CWM.LTough |
|  |  |

| CWM.LDMC | FDis_STR_ |
| --- | --- |
|  |  |

| **C) SPAD** |  |
| --- | --- |
| CWM.SPAD | FDis_SPAD_ |
|  |  |

| **D) WD** |  |
| --- | --- |
| CWM.WD | FDis_WD_ |
|  |  |

| **E) Tree height** |  |
| --- | --- |
| CWM.TH | FDis_TH_ |
|  |  |

| **F)** |  |
| --- | --- |
| FDis_ALL_ |  |
|  |  |
| **FIGURE S6.** Model validation plots for the final selected model (ΔDIC = 0; see Table 1) for each community functional attributes. For each attribute, the histogram on the left shows the posterior probability of a replicate of each observation and the plot on the right shows the relationship between the observed (x-axis) and the fitted (y-axis) values (i.e. overestimation and underestimation of the observed data by the model). A uniform distribution of posterior probabilities in the histogram would mean a well calibrated model (Lucas et al., 2020). | |

| Log(CWM_LA) | Log(CWM_SLA) | FDis_ACQ |
| --- | --- | --- |
|  |  |  |
|  |  |  |
| Log(CWM_Leaf Thickness) | CWM_Leaf Toughness | CWM_LDMC |
|  |  |  |
|  |  |  |
| FDis_STR | CWM_SPAD | FDis_SPAD |
|  |  |  |
|  |  |  |
| CWM_WD | FDis_WD |  |
|  |  |  |
|  |  |  |
| CWM_Tree height | FDis_Tree height | FDis_ALL |
|  |  |  |
|  |  |  |

**FIGURE S7.** Histogram of community functional attributes.

**TABLE S1**. Model comparison based on predictive performance (LPML, CPO failures). Env: Non-spatial model (Model E); Spa_Env: Spatial model (Model SE); LPML: Log Pseudo Marginal Likelihood (higher values indicate better predictive ability); CPO failures: Conditional Predictive Ordinates failures (values close to zero indicate reliable estimates).

| Community functional attributes | Model | LPML | CPO failures |
| --- | --- | --- | --- |
| CWM.LA | Env | 47.8 | 0 |
| CWM.LA | Spa_Env | 121.5 | 0 |
| CWM.SLA | Env | 100.5 | 0 |
| CWM.SLA | Spa_Env | 259.7 | 0 |
| FDis_ACQ | Env | 171.6 | 0 |
| FDis_ACQ | Spa_Env | 277.0 | 0 |
| CWM.Leaf Thickness | Env | 1477.5 | 0 |
| CWM.LeafThickness | Spa_Env | 1628.3 | 0 |
| CWM.Leaf Toughness | Env | 1521.7 | 0 |
| CWM.Leaf Toughness | Spa_Env | 1804.0 | 0 |
| CWM.LDMC | Env | 2003.9 | 0 |
| CWM.LDMC | Spa_Env | 2119.6 | 0 |
| FDis_STR | Env | 639.6 | 0 |
| FDis_STR | Spa_Env | 807.5 | 0 |
| CWM.SPAD | Env | -741.6 | 0 |
| CWM.SPAD | Spa_Env | -513.2 | 0 |
| FDis_SPAD | Env | 787.2 | 0 |
| FDis_SPAD | Spa_Env | 1028.2 | 0 |
| CWM.WD | Env | 2020.8 | 0 |
| CWM.WD | Spa_Env | 2265.0 | 0 |
| FDis_WD | Env | 552.5 | 0 |
| FDis_WD | Spa_Env | 940.4 | 0 |
| CWM.Tree height | Env | -1751.4 | 0 |
| CWM.Tree height | Spa_Env | -1466.8 | 0 |
| FDis_Tree height | Env | 892.2 | 0 |
| FDis_Tree height | Spa_Env | 1049.6 | 0 |
| FDis_ALL | Env | 206.7 | 0 |
| FDis_ALL | Spa_Env | 391.2 | 0 |

**TABLE S2**. Null model tests of trait–environment relationships along the topographic wetness index (TWI) gradient for selected traits. The observed slopes (β_obs_) from community-weighted mean (CWM) trait–TWI relationships were compared against null distributions generated by randomizing species trait values among species while preserving community composition. This null model framework tested whether the observed trait–environment relationships exceeded the expectations arising from environmentally structured species turnover. For each trait we report the observed slope (β_obs_), the mean and standard deviation of the null distribution, the standardized effect size (SES), and the permutation-based p-value. Significant SES values indicate environmentally structured trait variation beyond compositional turnover, whereas non-significant results indicate that the observed trait–environment relationship can be explained by species turnover along the environmental gradient. SES was calculated as: SES = (β_obs_ – mean(β_null_)) / sd(β_null_). See Appendix S4 for further details on the null model procedure.

| CWM Trait | β_obs_ | Mean(β_null_) | SD(β_null_) | SES | *P* value | Significant |
| --- | --- | --- | --- | --- | --- | --- |
| WD | -0.0026 | 1.00E-04 | 0.0031 | -0.8682 | 0.4184 | No |
| Tree height | -0.792 | -0.0053 | 0.3016 | -2.6079 | 0.006 | Yes |
| LDMC | -0.0032 | -1.00E-04 | 0.0057 | -0.5569 | 0.4014 | No |
| Leaf thickness | 0.0016 | -5.00E-04 | 0.0066 | 0.3254 | 0.8098 | No |
| SLA | -0.0114 | -0.0034 | 0.0309 | -0.2594 | 0.6977 | No |

A) TWI

B) P50

Figure 8 continued…

C) CV5m

D) Solar radiation

Figure 8 continued…

E) Fe

F) Mn

**FIGURE S8**. Null distributions of trait–environment slopes under environmentally structured species turnover for all environmental variables identified important in the spatial SE model (see Table 1). For each variable (A-F), histograms show the null distributions of slopes obtained by permuting species trait values among species while maintaining community composition unchanged. Each panel inside a variable block represents one trait. The vertical red line indicates the observed slope between the trait and a given trait relationship. If the observed slope falls outside the null distribution, the relationship indicates environmentally structured trait variation beyond species turnover. If the observed slope falls within the null distribution, the trait–environment association is consistent with species turnover along the environmental gradient. In our analysis, all traits, except tree height fall within the null expectations. Tree height in variable block A for TWI shows evidence of additional environmentally structured trait variation beyond compositional turnover. Hfeld: Tree height, Thick: Leaf thickness. See Appendix S4 for further details on the null model procedure.

| Community functional attributes | A) Kappa | B) Sigma | C) Range (m) |
| --- | --- | --- | --- |
| CWM.LA |  |  |  |
| CWM.SLA |  |  |  |
| FDis_ACQ_ |  |  |  |
| CWM.LThick |  |  |  |
| CWM.LTough |  |  |  |
| CWM.LDMC |  |  |  |
| FDis_STR_ |  |  |  |
| CWM.SPAD |  |  |  |
| FDis_SPAD_ |  |  |  |
| CWM.WD |  |  |  |
| FDis_WD_ |  |  |  |
| CWM.TH |  |  |  |
| FDis_TH_ |  |  |  |
| FDis_ALL_ |  |  |  |
| **FIGURE S9.** Spatial autocorrelation parameters, A) Kappa, B) variance and C) range, estimated by the best performing GLMs (see Table 1) for the community functional attributes, community-weighted mean (CWM) and functional dispersion (FDis). | | | |

**References**

Bunyavejchewin, S., Sinbumroong, A., Turner, B.L., & Davies, S.J. (2019) Natural disturbance and soils drive diversity and dynamics of seasonal dipterocarp forest in Southern Thailand *Journal of Tropical Ecology* 35: 95–107. https://doi.or/10.1017/s0266467419000075

Butterfield, B.J., & Callaway, R.M. (2013) A functional comparative approach to facilitation and its context dependence. *Functional Ecology*, 27(4), 907–917. https://doi.or/10.1111/1365-2435.12019

Chanthorn, W., Caughlin, T., Dechkla, S., & Brockelman, W.Y. (2013) The Relative Importance of Fungal Infection, Conspecific Density and Environmental Heterogeneity for Seedling Survival in a Dominant Tropical Tree *Biotropica* 45: 587–593. https://doi.or/10.1111/btp.12044

Chanthorn, W., Ratanapongsai, Y., Brockelman, W.Y., Allen, M.A., Favier, C., & Dubois, M.A. (2016) Viewing tropical forest succession as a three-dimensional dynamical system *Theoretical Ecology* 9: 163–172. https://doi.or/10.1007/s12080-015-0278-4

Chanthorn, W., Wiegand, T., Getzin, S., Brockelman, W.Y., & Nathalang, A. (2018) Spatial patterns of local species richness reveal importance of frugivores for tropical forest diversity. *Journal of Ecology*, 106(3), 925–935. https://doi.or/10.1111/1365-2745.12886

Chave, J., Coomes, D., Jansen, S., Lewis, S.L., Swenson, N.G., & Zanne, A.E. (2009) Towards a worldwide wood economics spectrum. *Ecology Letters*, 12(4), 351–366. https://doi.or/10.1111/j.1461-0248.2009.01285.x

Davies, S.J., Abiem, I., Abu Salim, K., Aguilar, S., Allen, D., Alonso, A., et al. (2021) ForestGEO: Understanding forest diversity and dynamics through a global observatory network. *Biological Conservation*, 253, 108907. https://doi.or/10.1016/j.biocon.2020.108907

Feldpausch, T.R., Banin, L., Phillips, O.L., Baker, T.R., Lewis, S.L., Quesada, C.A., et al. (2010) Height-diameter allometry of tropical forest trees. https://doi.or/10.5194/bgd-7-7727-2010

Jha, N., Tripathi, N.K., Chanthorn, W., Brockelman, W., Nathalang, A., Pélissier, R., et al. (2020) Forest aboveground biomass stock and resilience in a tropical landscape of Thailand. *Biogeosciences*, 17(1), 121–134. https://doi.or/10.5194/bg-17-121-2020

John, R., Dalling, J.W., Harms, K.E., Yavitt, J.B., Stallard, R.F., Mirabello, M., et al. (2007) Soil nutrients influence spatial distributions of tropical tree species. *Proceedings of the National Academy of Sciences*, 104(3), 864–869. https://doi.or/10.1073/pnas.0604666104

Kanagaraj, R., Wiegand, T., Comita, L.S., & Huth, A. (2011) Tropical tree species assemblages in topographical habitats change in time and with life stage *Journal of Ecology* 99: 1441–1452. https://doi.or/10.1111/j.1365-2745.2011.01878.x

Katabuchi, M. (2015) *LeafArea* : an R package for rapid digital image analysis of leaf area. *Ecological Research*, 30(6), 1073–1077. https://doi.or/10.1007/s11284-015-1307-x

Lucas, T., Python, A., & Redding, D. (2020) Graphical outputs and Spatial Cross-validation for the R-INLA package using INLAutils.

Paradinas, I., Illian, J., & Smout, S. (2022) Understanding spatial effects in species distribution models. *International Journal of Environmental & Earth Sciences*, 3(1), 1–4. https://doi.or/10.22541/au.164932203.31031660/v1

Pérez-Harguindeguy, N., Díaz, S., Garnier, E., Lavorel, S., Poorter, H., Jaureguiberry, P., et al. (2013) New handbook for standardised measurement of plant functional traits worldwide. *Australian Journal of Botany*, 61(3), 167. https://doi.or/10.1071/BT12225

Pothasin, P., Paradis, E., Brockelman, W.Y., Nathalang, A., Khemrugka, T., Lomwong, N., et al. (2022) Seed Size Variation of Trees and Lianas in a Tropical Forest of Southeast Asia: Allometry, Phylogeny, and Seed Trait - Plant Functional Trait Relationships. *Frontiers in Plant Science*, 13, 852167. https://doi.or/10.3389/fpls.2022.852167

Punchi-Manage, R., Getzin, S., Wiegand, T., Kanagaraj, R., Gunatilleke, C.V.S., Gunatilleke, I., et al. (2013) Effects of topography on structuring local species assemblages in a Sri Lankan mixed dipterocarp forest *Journal of Ecology* 101: 149–160. https://doi.or/10.1111/1365-2745.12017

Réjou‐Méchain, M., Tanguy, A., Piponiot, C., Chave, J., & Hérault, B. (2017) biomass : an r package for estimating above‐ground biomass and its uncertainty in tropical forests. *Methods in Ecology and Evolution*, 8(9), 1163–1167. https://doi.or/10.1111/2041-210X.12753
